# Return to Work Following Hip or Knee Arthroplasty: A One-Year Prospective Cohort Study in Participants with Direct Referral from Hospital to Occupational Health Care Services

**DOI:** 10.1007/s10926-024-10218-7

**Published:** 2024-06-19

**Authors:** Pauliina Kangas, Satu Soini, Konsta Pamilo, Visa Kervinen, Marja-Liisa Kinnunen

**Affiliations:** 1 Finnish Institute of Occupational Health, P.O. Box 40, 00032 Työterveyslaitos, Finland; 2 Coxa Hospital for Joint Replacement, Tampere, Finland; 3 Terveystalo Ltd, Occupational Health Services, Jyväskylä, Finland; 4 The Wellbeing Services County of Central Finland, Jyväskylä, Finland; 5 School of Medicine, University of Eastern Finland, Kuopio, Finland

**Keywords:** Return to work, Hip arthroplasty, Knee arthroplasty, Occupational health care, Sick leave, Workplace

## Abstract

**Purpose:**

In a new Finnish Coordinated Return to Work (CRTW) model, patients are referred to occupational health care after hip or knee arthroplasty. This study evaluated the CRTW model’s effect on return to work (RTW), activities used in occupational health care and in the workplace, and the patient- and work-related factors affecting early RTW.

**Methods:**

209 participants with occupational health care service underwent primary hip (THA) or total/unicondylar knee (KJA) arthroplasty and completed self-reported questionnaires after arthroplasty and at time of RTW. Factors affecting RTW, and the roles of occupational health care and the workplace in RTW were evaluated. Time to RTW was determined as days between the arthroplasty and RTW.

**Results:**

Mean time to RTW was 69 days after THA and 87 days after KJA. For easing RTW, work arrangements were made for 56% of the participants. The most utilized adjustments of work were enabling remote work and arranging limitations in work tasks. Participants with earlier RTW had lower physical workload, higher professional status and motivation to work, less pre-arthroplasty sick leave, and more positive personal expectations about the time to RTW compared to participants with later RTW (*p* < 0.001 for all). The linear regression and dominance analyses showed participants’ own expectations and pre-arthroplasty sick leave as the strongest factors affecting time to RTW.

**Conclusions:**

The CRTW model seems to shorten time to RTW after THA and KJA. Occupational health care and workplace play important roles in supporting RTW. Patients’ own expectations should be noted when giving pre-arthroplasty information.

## Introduction

Hip and knee osteoarthritis is a significant cause of disability and has a negative effect on work participation [1]. As the burden of osteoarthritis is expected to rise [2], the demand for hip and knee arthroplasty is predicted to increase in the Western countries [3–5]. In Finland, the number of hip and knee arthroplasty procedures has grown in the twenty-first century, especially among the working-age population: 30 to 40% of primary hip and knee arthroplasty is performed on patients aged under 65 years [6]. Thus, patients’ capacity of returning to work (RTW) following the arthroplasty must be noted. Although a fast-track protocol has been created to promote quick recovery from arthroplasty [7], recovery in fast-track studies has been measured from the perspective of the hospital (e.g. the length of stay in the hospital (LOS)) [8]. Although LOS is a valid metric, little is known about the recovery after fast-tracking in the longer term. Working capacity and RTW may better reflect overall recovery than LOS, especially from the patient's perspective.

Time to RTW after primary hip (THA) or total/unicondylar knee (KJA) arthroplasty varies between studies [9, 10]. In a Finnish cohort, 94% of patients returned to work within one year of THA, with a mean time of 103 days (from 10 to 354 days) [11]. Of the patients that underwent total knee arthroplasty, 87% returned to work within one year of the surgery, with a mean time of 116 days (from 28 to 356 days) [12]. However, even if the patients were able to return to work after an arthroplasty, work ability and productivity are commonly negatively affected, and this phenomenon still exists two years postoperatively [13].

Factors influencing RTW after THA or KJA have been evaluated in a few studies. Age, patient motivation, employment status before arthroplasty and type of job are important factors affecting the success of RTW [14]. Moreover, male gender, university education, working in business, finance or administration, and low physical demand in work appear to be associated to quick RTW after hip or knee arthroplasty [15]. Furthermore, a recent review concluded that patients being overweight or obese predict delayed RTW after hip arthroplasty [9]. In addition, psychosocial working conditions, such as more possibilities for personal job development, more work recognition and higher quality of supervisor leadership [16], as well as flexible working conditions [17, 18] have been associated with shorter time to RTW. However, little is known about the different actions made in workplaces or in occupational health care to facilitate RTW after arthroplasty.

Occupational health care has a strong role in the Finnish health care system. It is statutory for the employer to arrange occupational health care for all employees. The main tasks of this statutory occupational health care are preventing work-related health problems and work disability of employees. In addition, the employer may voluntarily arrange primary care services in occupational health care. According to the register of the Social Insurance Institute of Finland, the statutory occupational health care is realized in 90% of employees [19].

However, consulting an occupational health specialist has been rare after THA or KJA. Since surgeons are usually unfamiliar with the demands of patients’ work, the standard procedure has been to prescribe sick leave for two to three months. Thus, the length of the primary sick leave prescribed by surgeon has been quite similar for all patients, regardless of the nature of their work, and often the length of the sick leave has been impractical. To meet this challenge, a new co-operation procedure called the Coordinated Return to Work (CRTW) model has been developed between surgery clinics and occupational health care providers and is currently used in many districts in Finland. According to the CRTW model, the surgeons prescribe short sick leave, and the patient is systematically referred to occupational health care service. The recently published register-based Finnish study showed that the CRTW model shortens the time to RTW after THA and KJA [20].

The aim of this study is to evaluate how the CRTW model affects RTW after THA or KJA, and what kind of actions have been made in occupational health care and in workplace for supporting RTW. Furthermore, this study explores patient- and work-dependent factors influencing early RTW, and patients’ experiences of the CRTW model.

## Methods

### Coordinated Return to Work (CRTW) Model

The goal of the CRTW model is to systematically refer patients to occupational health care after arthroplasty in order to ensure efficient support to RTW. Referrals are made using an electronic referral system built between the information systems of hospital and occupational health care providers. If an electronic referral is not possible, the patients are instructed to contact their own occupational health care providers. The information of the CRTW model is given to the patients before arthroplasty. The surgeons at the hospital prescribe sick leave for one month, and the adequate total time to RTW is then assessed in occupational health care. The assessment is made individually, taking into account the work demands and the possibilities to adjust the workload. To support RTW, patients may receive guidance from an occupational physiotherapist. Furthermore, the occupational health specialist can refer patients to the different kind of medical and vocational rehabilitation after surgery. The most utilized rehabilitation interventions are physiotherapy, rehabilitation courses for the patients with arthritis, and work trials as part of vocational rehabilitation.

Since occupational health services are closely connected to the workplace, the actions supporting RTW are accomplished in collaboration with them and the employee. A joint negotiation, where the representatives of occupational health care and the employer participate together with the employee, is an important tool for assessing the adequate time to RTW and the adjustments in work needed for supporting RTW. An occupational physiotherapist often attends to the negotiation, assesses the physical workload and plans the needed adjustments. The adjustments typically include changes in working time, task limitations, work environment or to the physical demands of the work. Occupational health specialists follow the recovering process, ensure that rehabilitation options are appropriately utilized, and the work is modified when it is possible and necessary.

**Fig. 1:**
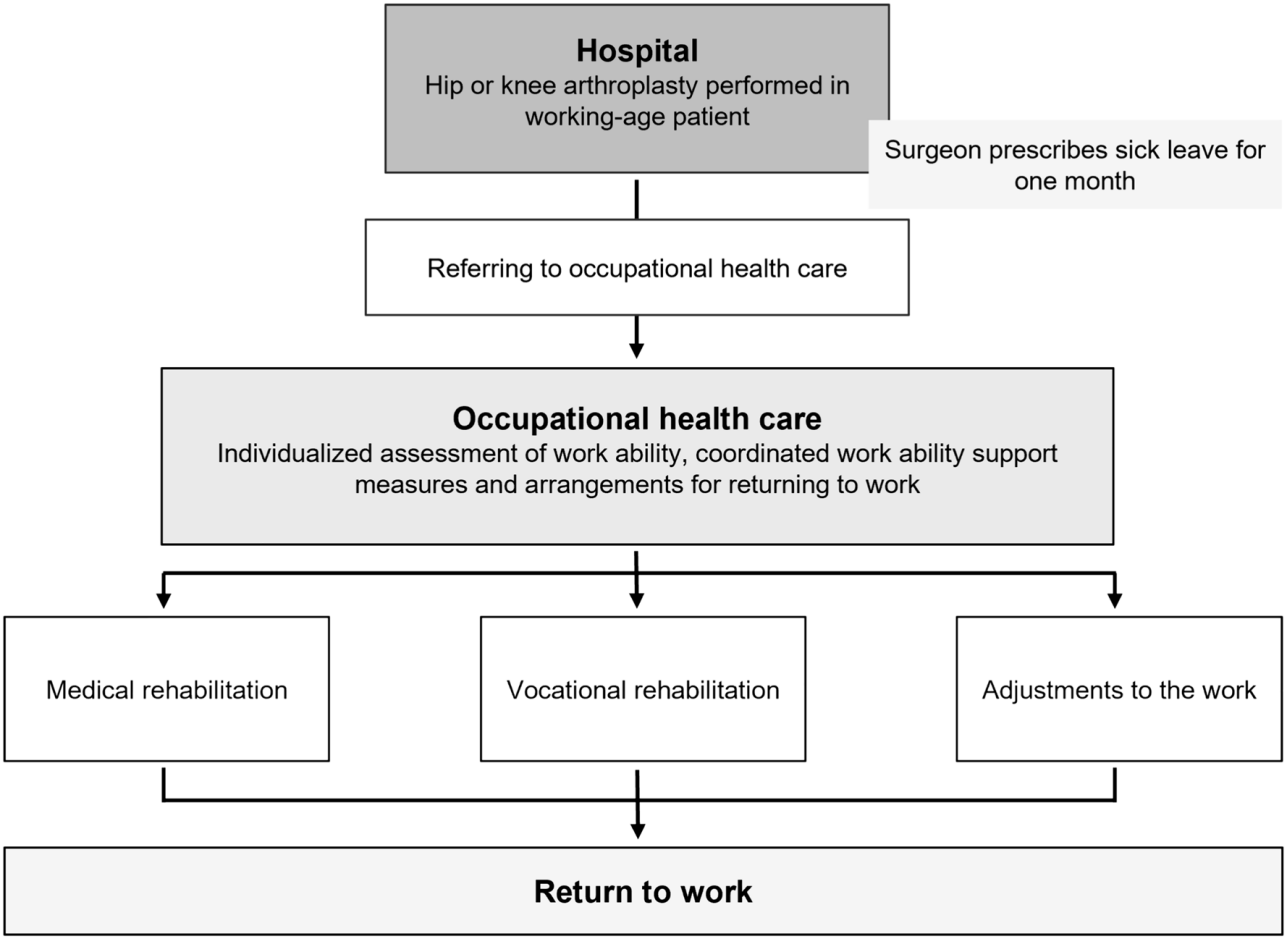
The principle of the CRTW model in THA or KJA patients

The CRTW model is performed as a part of statutory occupational health care, thus it concerns all employees undergoing hip or knee arthroplasty. The principle of the CRTW model is described in Fig. 1.

### Study Design and Population

The study participants were enrolled from patients that underwent THA or KJA at four hospitals across three districts in Finland. The hospitals varied from small local hospital to central hospital to large university hospitals. The inclusion criteria were: (1) THA or KJA (total or unicondylar) due to primary osteoarthritis performed between October 2020 and May 2021, (2) age 25 to 62 years, and (3) having a job and being entitled to use occupational health care services. All patients that fulfilled the inclusion criteria were informed of the study while recovering from the surgery at the hospital ward. Two self-reported questionnaires were given to the participants: a baseline questionnaire at the hospital ward, before discharging, and a post-arthroplasty questionnaire three months after the arthroplasty. The baseline questionnaire was given to the participants who gave written informed consent. After three months, they received either a link to the post-arthroplasty questionnaire by e-mail or a paper questionnaire by postal service. If the study participant had not returned to work by that time, they were requested to answer the questionnaire when RTW happens. The participants were reminded to answer twice. The participants who answered the questionnaire and returned to work within a year of the arthroplasty and unequivocally reported the day of RTW were included in the study.

### Self-Report Questionnaires

#### RTW

The day of RTW was asked in the follow-up questionnaire. The way of RTW (returning to full-time work with or without adjustments, on partial sickness allowance, or on work trials as part of vocational rehabilitation) was also asked. Time to RTW was determined as the number of days between the operation day and the self-reported day of returning to work. Returning to full-time and part-time work were both defined as successful RTW.

##### Actions in Occupational Health Care and in Workplace for Easing RTW

The follow-up questionnaire included questions on activities in occupational health care and in the workplace. These questions concerned the joint negotiations, and the discussions held between employees and the supervisors. Adjustments made in the workplace for easing RTW were asked in a multiple-choice question. Furthermore, the questions on rehabilitation were applied.

##### Patient-Related Factors that may Affect RTW

The baseline questionnaire included questions on participants’ background characteristics, such as age, sex, educational level (low = primary school; intermediate = secondary school and/or 2–3 years of specialization; high = bachelor’s or higher level of education), and medical history (underlying diseases yes/no). Moreover, participants’ own expectations of RTW and working status prior to the arthroplasty were questioned at the baseline.

The Oxford Hip Score and the Oxford Knee Score self-report questionnaires were used to assess physical functioning, pain, and limitation due to physical problems [21–23]. Twelve questions included to both questionnaires, and the response options ranged from zero to four, where zero represented the worst outcome and four represented the best. Thus, total points ranged from 0 to 48 points. Oxford scores were evaluated both in baseline and follow-up questionnaires.

##### Work-Related Factors that may Affect RTW

Work-related factors, including the size of the workplace, physical and psychosocial demands of the work, participant’s motivation to the work, job position, and working status prior the arthroplasty (in work, on sick leave, or in some other arrangement) were asked at the baseline. Physical demands of the work were examined by questions regarding whole body physical exertion; repeated work movements; standing; bent, twisted or uncomfortable postures of the spine; continuous moving/walking; lifting, holding, or carrying by hands; sitting still; sitting on the knees or squatting; holding hands above shoulder level; standing or sitting on a vibrating platform; or working in cold conditions (under 10 C°). Each question was scored from 0 to 2 (0 = no such loading factor in the work; 1 = the loading factor occurs in the work to some extent; 2 = the loading factor occurs in the work a lot). Total physical load in work was calculated as a sum of the points of each separate question. The questionnaire of physical workload was modified from the TIKKA workload assessment tool [24].

##### Participants’ Experiences of the CRTW Model

Participants’ opinion of the new CRTW model were asked at the follow-up. It was an open-ended question “What do you think about the protocol, where an orthopedic surgeon prescribes 2 to 4 weeks sick leave, and the addition of the sick leave and the RTW is coordinated in the occupational health services? What is good about it, what should improve?”. Furthermore, a question was applied to evaluate how participants experienced the timing of the RTW.

### Statistical Analyses

The information about RTW was reported as descriptive data. Time between the day of arthroplasty and the day of RTW was calculated, and the results were given as mean and standard deviation (SD). Furthermore, the medians were calculated for the analyses mentioned below. Percentages of different ways of RTW (returning to full-time work with or without adjustments, on partial sickness allowance, or on work trials as part of vocational rehabilitation) as well as working status immediately before arthroplasty were reported.

Actions in occupational health care and in workplace for easing RTW were reported as percentages of participants who had discussed of the RTW with their supervisor, and who had participated in the joint negotiations in occupational health care. Percentage of the participants that experienced sufficient guidance for rehabilitation was reported. The adjustments made in the workplace for easing RTW were reported as percentages and visualized in Fig. 3.

For analyzing the patient- and work-related factors affecting time to RTW, the study population was divided into the two groups: participants having time to RTW shorter than median (*n* = 101), and participants having time to RTW median or longer (*n* = 108). Since the time to RTW differs between THA and KJA, the median value was determined separately based on the operated joint (hip or knee). The characteristics between the groups were compared using an independent samples t-test in cases of continuous variables. A Pearson’s Chi-square test or Fisher-Freeman-Halton exact test was used in cases of categorical variables. Continuous variables with normal distribution were reported as means and standard deviations, categorical variables were reported as number of cases and percentages.

The factors affecting time to RTW were further analyzed by linear regression with Enter method. To compare the relative importance of different factors that affect time to RTW, a dominance analysis was carried out [25]. The linear regression and the dominance analyses were carried out for the whole study population, and separately for participants underwent THA and KJA.

Participants’ experiences of the CRTW model were analyzed from the qualitative data from the open-ended question. The results were reported in descriptive ways. The results of the multiple-choice question: “Do you think that the RTW happened timely?” were reported as proportions of the answers “yes/no/can’t express”.

**Fig. 2:**
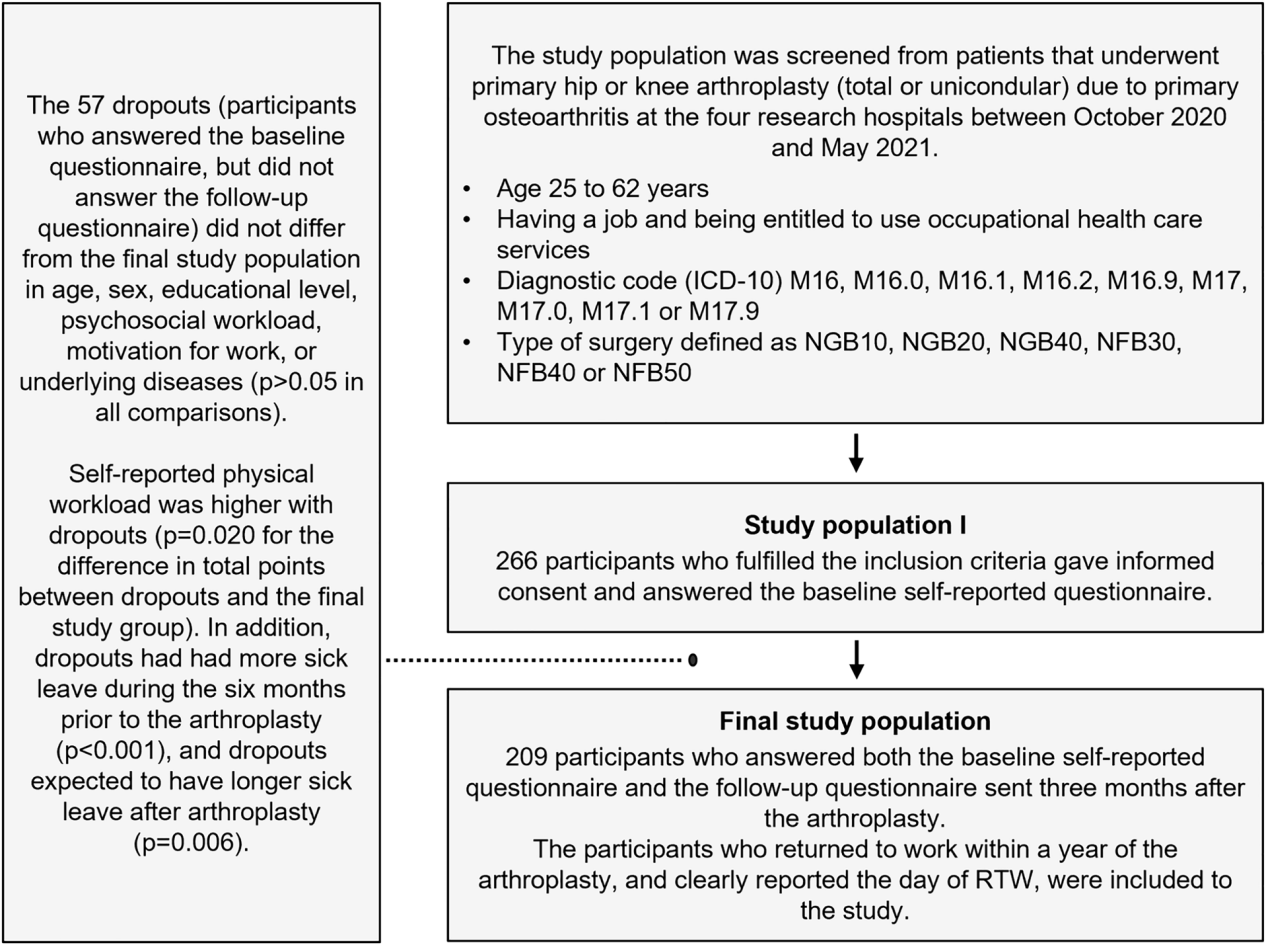
Study participants and the differences between dropouts and the final study population

The differences between the final study population and dropouts (Fig. 2) were analyzed using an independent samples t-test in cases of continuous variables, and a Pearson’s Chi-square test or Fisher-Freeman-Halton exact test in cases of categorical variables.

All testing was two-sided, and *p*-values < 0.05 were considered as statistically significant. The data were analyzed using SPSS version 27 (IBM Corp, Armonk, NY), except the dominance analyses, which were analyzed using Stata Statistical Software version 18 (StataCorp, College Station, Texas, USA).

## Results

### Participants and RTW

The study participants were enrolled from 638 working-aged patients that underwent THA or KJA at the study hospitals. The exact number of the patients that were entitled to use occupational health care is unknown, but based on the Finnish employment rate and the coverage of occupational health care services [19, 26], approximately 60%, i.e., about 380 patients were in the target group of the study. 266 participants out of the 380 (70%) filled out the baseline questionnaire. 57 of them did not answer to the follow-up questionnaire. Thus, the final study group consisted of 209 participants. The study participants and the description of the dropouts are represented in Fig. 2.

A total of 94 study subjects underwent THA, and 115 study subjects underwent KJA. The mean time of RTW was 68 days (SD 32) after THA, and the mean time of RTW after KJA was 87 days (SD 52). Sixty-eight percent returned straight to the full-time work (with or without some adjustments), 22% returned to work on partial sickness allowance, 3% of participants began work trials as part of vocational rehabilitation, and 7% had some other arrangements. Immediately prior to the arthroplasty, 77% of the participants were in work, 13% were on sick leave, 4% had partial economic integration of the handicapped, 3% were on partial sickness allowance, and 4% had some other arrangement such as study leave or furlough. At the time of arthroplasty, the patients reported the average pain in the last four weeks as very mild in 4% of cases, mild in 10% of cases, moderate in 59% of cases, and severe in 28% of cases, while none was painless. At the time of RTW, the corresponding figures were 22% experiencing no pain, 31% with very mild pain, 26% mild pain, 20% moderate pain, and 1% severe pain. In the Oxford score questionnaires, with the participants that underwent hip arthroplasty, mean total points were 22 (ranged from 8 to 43) before arthroplasty, and 35 points (ranged from 19 to 48) at the time of RTW. With the participants that underwent knee arthroplasty, mean total points were 25 (ranged from 10 to 46) before arthroplasty, and 41 points (ranged from 19 to 48) at the time of RTW.

### Actions in Occupational Health Care and in Workplace for Easing RTW

**Fig. 3:**
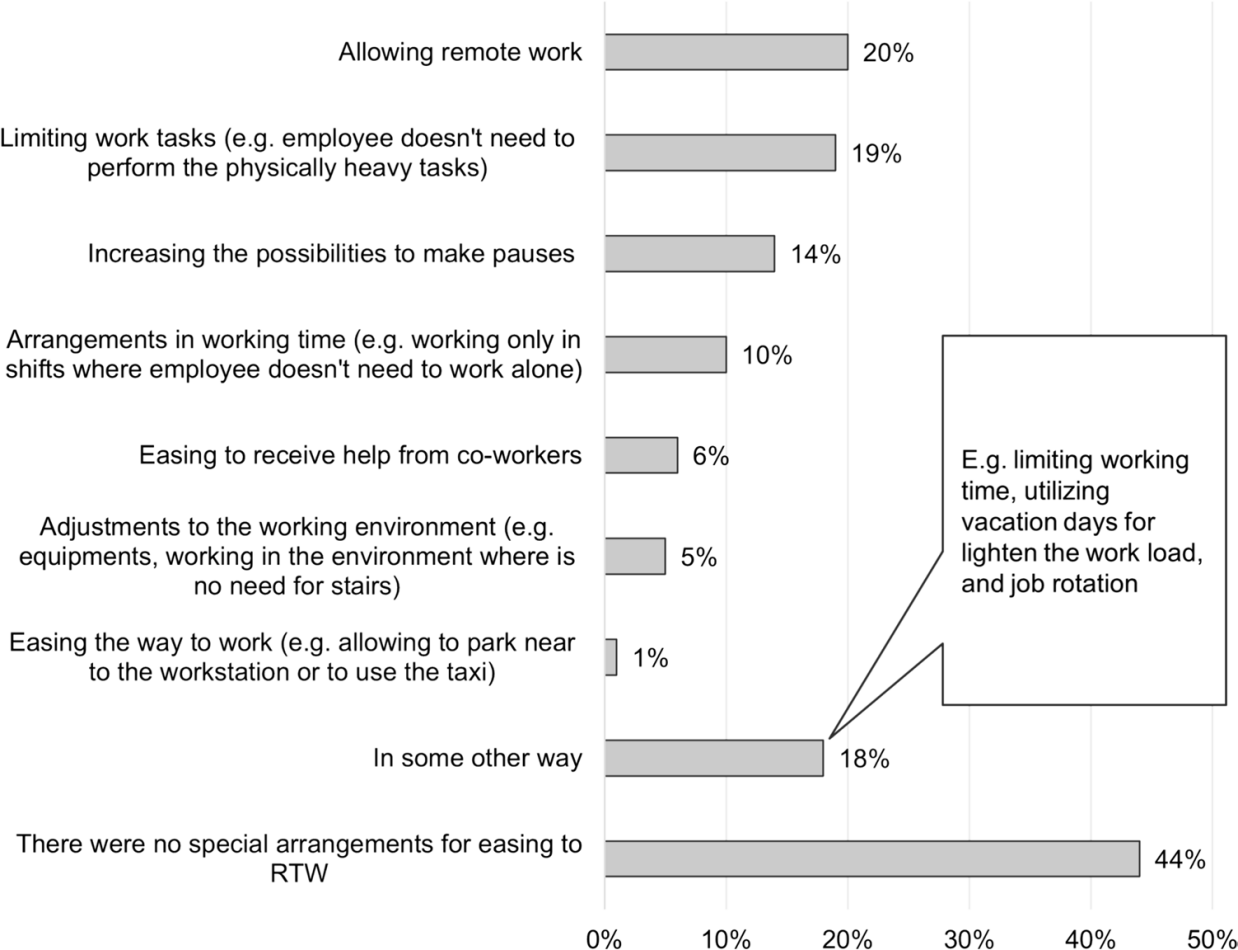
Used adjustments to work and working conditions for easing RTW, displaying the percentage of participants who chose the category. The respondents were able to choose more than one option. Number of answers: 324, number of respondents: 209

Seventy-five percent of participants had discussed RTW with their supervisor after arthroplasty. A joint negotiation, where the representatives of occupational health care and the employer participate together with the employee, was arranged in 51% of cases. In the joint negotiations, different rehabilitation arrangements and adjustments to the employee’s work and working conditions were planned for targeting smooth RTW. In some cases, adjustments to the work could have been made without a joint negotiation. Eighty percent of participants reported that they had received sufficient guidance for rehabilitation. Fourteen percent reported that the guidance was not enough, and 6% of participants could not express their answer to the question. Multiple adjustments were made in the workplace for easing RTW. Some arrangement was made for 56% of the participants. The most utilized adjustment was enabling remote work (20% of the participants). The detailed adjustments used for supporting RTW are represented in Fig. 3.

### Patient-Related Factors Associating with the Time to RTW

The general characteristics of the study population are presented in Table 1 in two groups: 1. subjects who returned to work before the median time (*n* = 101), and 2. Subjects who returned to work in median time or later (*N* = 108). The median time to RTW was 63 days after THA and 79 days after KJA.

**Table 1:** Characteristics of the study population, stratified by time to RTW

Characteristics	Time to RTW is shorter than median (*N* = 101)			Time to RTW is median or longer (*N* = 108)			*P*-value
	N	%	Mean ± SD	N	%	Mean ± SD	
Age (years)			55 ± 5			56 ± 5	0.121
Sex							0.084
Female	57	56%		72	67%		
Male	44	44%		36	33%		
Educational level^a^							0.076
Low	7	7%		15	14%		
Intermediated	49	49%		58	54%		
High	43	43%		32	30%		
Missing	2	2%		3	3%		
Underlying diseases							0.230
Yes	66	65%		64	59%		
No	33	33%		40	37%		
Can’t express	0	0%		3	3%		
Missing	2	2%		1	1%		
Sick leave before arthroplasty (because of hip or knee osteoarthritis)							< 0.001
0–5 days	85	84%		61	56%		
6–20 days	7	7%		21	19%		
21–60 days	5	5%		14	13%		
60–90 days	2	2%		4	4%		
Over 90 days	0	0%		7	6%		
Missing	2	2%		1	1%		
Own expectation about the time to RTW after arthroplasty							< 0.001
2 to 8 weeks	54	53%		17	16%		
8 to 16 weeks	39	39%		67	62%		
16 weeks or not expecting to RTW	4	4%		20	19%		
Missing	4	4%		4	4%		
Self-reported average pain in the last four weeks before arthroplasty							0.400
No pain	0	0%		0	0%		
Very mild pain	5	5%		2	2%		
Mild pain	10	10%		9	9%		
Moderate pain	59	58%		56	52%		
Severe pain	22	22%		32	29%		
Missing	5	5%		9	8%		

In the grouping, the median value was determined separately based on the operated joint (hip or knee). SD = standard deviation. ^a^Low = primary school; intermediate = secondary school and/or 2–3 years of specialization; high = bachelor’s or higher level of education. *P*-values denote differences between shorter time to RTW and longer time to RTW

The groups did not differ in age, sex, underlying diseases, and in self-reported pre-arthroplasty pain (*p* > 0.05 for all) (Table 1). Educational level (low, intermediate, high) did not differ between the groups with shorter and longer time to RTW (*p* = 0.076). However, when evaluating mean time to RTW with only two educational groups (participants having a bachelor’s or higher level of education and participants having a lower educational level), the former had significantly shorter time to RTW (mean 67 versus 85 days, *p* = 0.005). Participants with time to RTW shorter than the median had less pre-arthroplasty sick leave, and more positive personal expectations about the time to RTW compared to the group having median or longer time to RTW (*p* < 0.001 for both) (Table 1).

### Work-Related Factors Associating with Time to RTW

Work-related characteristics are presented in Tables 2 and 3 in two groups: 1. subjects who returned to work before the median time (*n* = 101), and 2. subjects who returned to work in median time or later (*N* = 108).

**Table 2:** Work-related characteristics, stratified by time to RTW

Characteristics	Time to RTW is shorter than median (*N* = 101)			Time to RTW is median or longer (*N* = 108)			*P*-value
	N	%	Mean ± SD	N	%	Mean ± SD	
Professional status^a^							< 0.001
Worker	35	35%		77	71%		
Upper clerical worker, expert	28	28%		16	15%		
Middle or upper management	16	16%		2	2%		
Supervisor	10	10%		5	5%		
Entrepreneur	3	3%		2	2%		
Missing	9	9%		6	6%		
Workplace size							0.179
Under 10 employees	9	9%		4	4%		
10–20 employees	9	9%		13	12%		
21–50 employees	9	9%		15	14%		
51–250 employees	23	23%		16	15%		
Over 250 employees	46	46%		58	54%		
Missing	5	5%		2	2%		
Self-reported motivation to the work							< 0.001
Highly motivated	66	65%		38	35%		
Quite motivated	28	29%		60	56%		
Can’t express	4	4%		3	3%		
Quite poorly motivated	3	3%		4	4%		
Very poorly motivated	0	0%		3	3%		
Self-reported psychosocial load of the work							0.095
Light psychosocial load	0	0%		4	4%		
Quite light psychosocial load	4	4%		5	5%		
Somewhat psychosocial load	28	28%		32	30%		
Quite much psychosocial load	42	42%		53	50%		
Very much psychosocial load	24	24%		14	13%		
Can’t express	1	1%		0	0%		
Missing	2	2%		0	0%		

In the grouping, the median value was determined separately based on the operated joint (hip or knee). SD = standard deviation. ^a^10 participants had chosen more than one options to the question of professional status (for example, both upper clerical worker/expert and supervisor). For the sake of clarity, these cases were excluded from the statistical analysis and are represented as “missing” in the table. *P*-values denote differences between shorter time to RTW and longer time to RTW

**Table 3:** Self-reported physical workload, stratified by the time to RTW

Characteristics	Time to RTW is shorter than median (*N* = 101)			Time to RTW is median or longer (*N* = 108)			*P*-value
	N	%	Mean ± SD	N	%	Mean ± SD	
Self-reported physical workload							
Whole body physical exertion							< 0.001
None (0)	64	63%		31	29%		
Some (1)	21	21%		52	48%		
A lot (2)	13	13%		23	21%		
Missing	3	3%		2	2%		
Repeated work movements							0.352
None (0)	15	15%		11	10%		
Some (1)	46	46%		44	41%		
A lot (2)	39	39%		51	47%		
Missing	1	1%		2	2%		
Standing still							0.026
None (0)	22	22%		15	14%		
Some (1)	64	63%		59	55%		
A lot (2)	14	14%		30	28%		
Missing	1	1%		4	4%		
Bent, twisted or uncomfortable postures of the spine							< 0.001
None (0)	50	50%		24	22%		
Some (1)	37	37%		48	44%		
A lot (2)	12	12%		35	32%		
Missing	2	2%		1	1%		
Continuous moving/walking							0.001
None (0)	23	23%		13	12%		
Some (1)	49	49%		38	35%		
A lot (2)	26	26%		56	52%		
Missing	3	3%		1	1%		
Lifting, holding, or carrying by hands							< 0.001
None (0)	40	40%		25	23%		
Some (1)	47	47%		47	44%		
A lot (2)	11	11%		35	32%		
Missing	3	3%		1	1%		
Sitting still							< 0.001
None (0)	3	3%		17	16%		
Some (1)	32	32%		51	47%		
A lot (2)	65	64%		38	35%		
Missing	1	1%		2	2%		
Sitting on the knees or squatting							< 0.001
None (0)	53	53%		24	22%		
Some (1)	35	35%		59	55%		
A lot (2)	10	10%		21	19%		
Missing	3	3%		4	4%		
Holding hands above shoulder level							< 0.001
None (0)	63	62%		31	29%		
Some (1)	25	25%		62	57%		
A lot (2)	11	11%		12	11%		
Missing	2	2%		3	3%		
Standing or sitting on a vibrating platform							0.557
None (0)	86	85%		95	88%		
Some (1)	6	6%		10	9%		
A lot (2)	5	5%		3	3%		
Missing	4	4%		0	0%		
Working in the cold conditions (under 10 C°)							0.096
None (0)	74	73%		65	60%		
Some (1)	21	21%		34	32%		
A lot (2)	4	4%		8	7%		
Missing	2	2%		1	1%		
Total points for physical load			8 ± 4			11 ± 4	< 0.001

In the grouping, the median value was determined separately based on the operated joint (hip or knee). SD = standard deviation. *P*-values denote differences between shorter time to RTW and longer time to RTW

Professional status as well as self-reported motivation to the work was higher in the group with shorter time to RTW (*p* < 0.001) (Table 2). In the group where time to RTW was shorter, physical workload was lower than in the group where time to RTW was longer (*p* < 0.001 for the difference in total points for physical workload) (Table 3). Besides the total physical workload, the group with longer time to RTW, had also more whole body physical exertion, standing still, bent, twisted or uncomfortable postures, continuous moving/walking, lifting, holding, or carrying by hands, sitting on the knees or squatting, and holding hands above shoulder level, and less sitting still (*p* < 0.05 for all)(Table 3). Psychosocial workload or size of the workplace did not differ according to time to RTW (*p* = 0.095 and *p* = 0.179, respectively) (Table 2).

### The Strength of the Factors Associated with Time to RTW

Factors associating time to RTW after arthroplasty were also evaluated in a linear regression model and in a dominance analysis (Tables 4, 5, 6). The model included age, sex, physical workload, underlying diseases (yes or no), patient’s own expectation about time to RTW, educational level (low, intermediate, high), sick leave during the six months before the arthroplasty (because of hip or knee osteoarthritis), patient-reported average pain in the last four weeks before the arthroplasty, patient’s motivation in their work, and psychosocial load of the work. The analysis was performed for the whole study population, and in addition, separately for the participants that underwent THA and KJA.

**Table 4:** Linear regression and dominance analysis of variables associated with time to RTW. Participants that underwent THA or KJA are included in the model

	B	Beta	95% Confidence interval for B		*P*-value	Relative importance of the predicting variable (Ranks in the dominance analysis)
			Lower bound	Upper bound		
Patient’s own expectation about time to RTW	27.441	0.382	15.862	39.019	** < 0.001**	1
Sick leave during 6 months before arthroplasty	12.398	0.272	5.830	18.967	** < 0.001**	2
Physical workload	0.848	0.077	−0.902	2.599	0.340	3
Age	0.752	0.075	−0.588	2.092	0.269	4
Patient’s motivation to the work	−1.218	−0.022	−8.986	6.550	0.757	5
Patient-reported average pain in the last 4 weeks before arthroplasty	−2.020	−0.031	−11.216	7.177	0.665	6
Educational level (low, intermediate, high)	0.381	0.005	−10.498	11.261	0.945	7
Sex	4.450	0.047	−8.553	17.453	0.500	8
Underlying disease (yes or no)	−1.890	−0.020	−14.487	10.707	0.767	9
Psychosocial load of the work	2.086	0.039	−5.137	9.310	0.569	10

Enter method is used in the linear regression analysis. R square of the model is 0.372, adjusted R square is 0.330. Durbin-Watson 1.293, Anova < 0.001. Male = 0, female = 1. Underlying disease: yes = 1, no = 0

**Table 5:** Linear regression and dominance analysis of variables associated with time to RTW. Participants that underwent THA are included in the model

	B	Beta	95% Confidence interval for B		*P*-value	Relative importance of the predicting variable (Ranks in the dominance analysis)
			Lower bound	Upper bound		
Sick leave during 6 months before arthroplasty	10.965	0.337	3.917	18.012	**0.003**	1
Patient’s own expectation about time to RTW	12.896	0.244	0.218	25.573	**0.046**	2
Physical workload	1.152	0.151	−0.577	2.881	0.188	3
Sex	14.364	0.218	0.749	27.979	**0.039**	4
Patient-reported average pain in the last 4 weeks before arthroplasty (4 = no pain, 0 = severe pain)	−2.797	−0.067	−11.807	6.213	0.537	5
Age	0.899	0.142	−0.358	2.155	0.158	6
Educational level (low, intermediate, high)	−3.864	−0.073	−14.978	7.249	0.489	7
Underlying disease (yes or no)	8.775	0.133	−4.469	22.019	0.190	8
Patient’s motivation to the work	−1.603	−0.036	−10.442	7.235	0.718	9
Psychosocial load of the work	2.653	0.073	−4.827	10.132	0.481	10

Enter method is used in the linear regression analysis. R square of the model is 0.487, adjusted R square is 0.400. Durbin-Watson 1.791, Anova < 0.001. Male = 0, female = 1. Underlying disease yes = 1, no = 0

**Table 6:** Linear regression and dominance analysis of variables associated with time to RTW. Participants that underwent KJA are included in the model

	B	Beta	95% Confidence interval for B		*P*-value	Relative importance of the predicting variable (Ranks in the dominance analysis)
			Lower bound	Upper bound		
Patient’s own expectation about time to RTW	35.406	0.428	17.130	53.681	** < 0.001**	1
Sick leave during 6 months before arthroplasty	15.601	0.300	5.237	25.965	**0.004**	2
Physical workload	0.722	0.055	−2.301	3.745	0.636	3
Underlying disease (yes or no)	−11.502	−0.101	−32.362	9.359	0.276	4
Patient’s motivation to the work	−1.307	−0.022	−13.103	10.490	0.826	5
Educational level (low, intermediate, high)	8.921	0.103	−9.540	27.381	0.339	6
Patient-reported average pain in the last 4 weeks before arthroplasty (4 = no pain, 0 = severe pain)	−2.852	−0.035	−19.452	13.747	0.733	7
Age	0.409	0.030	−2.159	2.977	0.752	8
Sex	−5.093	−0.046	−26.283	16.097	0.634	9
Psychosocial load of the work	−0.153	−0.002	−11.996	11.689	0.979	10

Enter method is used in the linear regression analysis. R square of the model is 0.380, adjusted R square is 0.301. Durbin-Watson 1.243, Anova < 0.001. Male = 0, female = 1. Underlying disease yes = 1, no = 0

In the analysis performed for the whole study population (Table 4), patient’s own expectation about time to RTW and sick leave before arthroplasty were statistically significantly predicting time to RTW (*p* < 0.001 for both). The dominance analysis revealed that the strongest factor in determining time to RTW was the patient’s own expectation, and the second strongest factor was the sick leave before arthroplasty.

When the participants that underwent THA were in focus (Table 5), sick leave before arthroplasty, patient’s own expectation, and the female sex all predicted time to RTW (*p* < 0.05 for all). The strongest factor in determining time to RTW was the sick leave before arthroplasty, and the second strongest was the patient’s own expectation.

Among the participants that underwent KJA (Table 6), patient’s own expectation and sick leave before arthroplasty both predicted time to RTW (*p* < 0.01 for both), the former being the strongest to determine time to RTW, while the latter was the second strongest predictor.

### Participants’ Experiences about the CRTW Model

Seventy-seven percent of the participants reported that the RTW occurred in a timely manner. Nine percent reported that the timing was not suitable, and 14% could not say for sure. 191/209 participants answered an open-ended question about the CRTW model. Approximately, two-thirds of participants’ opinions of the new model were positive, describing the benefits of familiar services and regular controls in the occupational health services, the individualized evaluation of the work ability based on workload, and the possibilities to adjust their work. The majority of the negative comments concerned the experiences of too short sick leave prescribed by the surgeon, and the inconvenience of being forced to repeatedly visit physicians.

## Discussion

### The Main Findings of the Study

The most important findings of this study are: (1) After the implementation of the CRTW, mean time to RWT following THA or KJA had shortened 33% and 25% compared to the times documented in previous Finnish cohorts [11, 12]; (2) Due to the actions of occupational health care and the employers, multiple arrangements were made for supporting RTW after arthroplasty; (3) The most important factors influencing time to RTW were the patients’ own expectations for the length of sick leave after arthroplasty, and the sick leave during the six months before the arthroplasty (because of hip or knee osteoarthritis); (4) Participants’ experiences about the CRTW model were mostly positive.

### The Role of the CRTW Model in Shortening Time to RTW

According to this study, it can be inferred that time to RTW after arthroplasty can be shortened by referring patients to occupational health care services at orthopedic clinics. This finding is in good concordance with the results of the recent register-based Finnish study [20], albeit in that study, the effect of the CRTW model on time to RTW after THA and KJA was somewhat smaller than in the current study. In that register-based study, the study population consisted of the general working-age population including unemployed subjects and employed subjects without occupational health care services. Thus, the CRTW model was not utilized in the whole study population, and this may had decreased the observed effects on the time to RTW. However, the findings of both studies emphasize the importance of collaboration between orthopedic clinics and occupational health care services. In occupational health care services, post-arthroplasty sick leave can be individually determined, considering not only the operation the patient is recovering from, but also the workload and the possibilities to adjust the workload. When assessing work ability, it is important to assess the workload in conjunction with the capacity of the subject. This is possible in occupational health care services, which are closely connected to workplaces. When the appropriate sick leave after THA and KJA is individually assessed, as is done in CRTW model, time to RTW can more accurately be used as an indicator of patients’ postoperative recovery. The role of occupational health care in supporting RTW after arthroplasty has rarely been evaluated previously. It has been speculated that collaboration between occupational physicians and surgeons in orthopedic clinics may lead to increased and earlier ability to work after knee arthroplasty [27], and some preliminary evidence of this has already been reported [28] prior to the present study. However, consulting an occupational health specialist within three months after knee arthroplasty did not result in shorter time to RTW among 182 patients [29]. The involvement of occupational health staff was noted as one of the patient-reported factors associated with a positive experience of RTW [18]. In addition, employers have evaluated occupational health services as an important partner in supporting employee’s RTW after arthroplasty [30].

### Supporting RTW in Collaboration with Occupational Health Care and Workplace

Some previous studies have underlined the significance of actions made in the workplace to support RTW after arthroplasty. According to employees, workplace support and adaptation of their job play important roles in easing RTW [18]. In the interviews of twenty-five workplace representatives, employers were motivated to support employees to RTW after arthroplasty, and changes to the work, such as modifying work tasks or working time, providing additional or adapted equipment and furniture, and colleague support, were used to ease RTW [30]. In the current study, multiple actions were made in workplaces to support RTW after arthroplasty. Without systematically referring patients to occupational health care at the time of arthroplasty, thus utilizing the new CRTW model, the actions made in workplaces for supporting to RTW would not had been as numerous or timely. Without the CRTW model, information about the arthroplasty procedures may not reach occupational health specialist in a timely manner, and the supporting RTW may not happen or may be delayed. Employer motivation and readiness to make arrangements for easing RTW are crucial for successful outcomes. Thus, it is important to enlighten employers and give them appropriate information about supporting employees after arthroplasty. Good collaboration between occupational health care and the workplace contributes to this understanding.

### Patient- and Work-Related Factors Affecting Time to RTW

In the current study, we examined different patient and work-related factors associated with early RTW after arthroplasty. In the group where time to RTW was shorter than the median, physical workload was lower, self-reported motivation to the work was higher, and professional status was higher compared to the group with median or longer times to RTW. These findings are consistent with previous studies [14, 15]. In this study, sick leave during the six months before arthroplasty was associated with delayed time to RTW. If the participant’s preceding sick leave had been over 90 days, the mean time to RTW after arthroplasty was over twice as long as the average time. It can be expected that the participants experiencing more severe symptoms before arthroplasty and difficulties to stay in work will also need longer postoperative sick leave. However, this finding emphasizes supporting employees both before and after arthroplasty. Adjustments to the work and work environment may therefore be needed prior to arthroplasty. Moreover, this finding raises a question whether time to RTW after arthroplasty can be shortened doing the operation earlier for these patients.

Together with the sick leave before arthroplasty, patients’ own expectations of the length of sick leave was noted as the most important factor associated with time to RTW. In a previous study, patient’s expectation of the length of sick leave was a predictor for not returning to work after knee arthroplasty [31]. Furthermore, patients’ expectations have noticed to been prognostic for dissatisfaction with performing work-related knee-straining activities after total knee arthroplasty [32]. In the current study, it is notable that patients’ own expectations was the most important factor determining time to RTW when observing the whole study population (both THA and KJA patients), and when observing only patients that underwent KJA. With THA patients, the sick leave before arthroplasty was observed to be the most significant factor, while the patient’s own expectations was the second most significant factor. The expectation was a strong factor affecting time to RTW, even if age, sex, physical and psychosocial workload, underlying diseases, educational level, sick leave during the six months before arthroplasty, patient-reported average pain in the last four weeks before arthroplasty, and patient’s motivation to the work were included to the model. This finding is novel and significant and emphasizes ensuring patients are well advised over the course of the entire arthroplasty process. As the patients’ own expectations are such a strong factor, it is important that patients are told the possibilities of rehabilitation and arrangements in the workplace for easing RTW after the operation. This advising should be done well before arthroplasty, and by both occupational health specialist and orthopedic surgeons.

### The Limitations of the Study

This study has some limitations, especially when it comes to evaluating sick leave after arthroplasty. Time to RTW was calculated from the day when participants reported they started work (full-time or part-time). It is possible that the period from the day of arthroplasty to RTW has included vacation days in addition to sick leave prescribed by the physician. The participants answered the baseline questionnaire at the hospital ward while recovering from the surgery. Therefore, it is possible that success of the surgery or patient’s pain level may had an influence on the answers about the patient’s own expectations for the length of sick leave after the arthroplasty. On the other hand, the dropouts, the participants who answered the baseline questionnaire, but did not answer the follow-up questionnaire, had higher physical workload, had more sick leave during the six months before arthroplasty, and expected to have longer sick leave after arthroplasty. Thus, time to RTW after arthroplasty may have been longer in that group compared to the final study population. In the study population, time to RTW after KJA was significantly lower than in the previous study in Finnish population [12], but it should be noticed that the previous study concerned only patients who had undergone total knee arthroplasty, while in the current study both total and unicondylar knee arthroplasty were included. However, in the population of the current study, approximately less than ten percent of the patients that underwent knee arthroplasty, had unicondylar arthroplasty. Therefore, it may assume that this hardly had an important effect on the study results. Thus, even if these limits are taken into account, the results of this study indicate that time to RTW after THA or KJA has shortened after the implementation of the CRTW model. More studies, especially register-based studies, are needed to evaluate the effect of the CRTW model more accurately on sick leave.

### The Strengths of the Study

The strength of this study is its representation of a comprehensive evaluation of the influences of the CRTW model. New information was gathered about RTW, particularly the numerous actions made in occupational health care and workplace for easing RTW after THA or KJA. Furthermore, this study demonstrated factors predicting shorter time to RTW after arthroplasty. The main advance of the CRTW model is the individualized assessment of work ability based on not only patient performance but also the workload and the possibilities to adjust their work. When physicians in occupational health care become more experienced with the new model, the common healing process after arthroplasty, and RTW (especially in physically demanding work), sick leaves after arthroplasty may further shorten. Encompassing the electronic referral system from hospitals to occupational health care services will further enhance the influence of the CRTW model.

## Conclusion

Referring patient from orthopedic unit to occupational health care after THA and KJA shortens the time to RTW. Occupational health care and the workplace play an important role in supporting RTW, and many kinds of adjustments are done in the workplace for easing RTW. The strongest factors in determining time to RTW after arthroplasty were the patient’s own expectation about time to RTW and the sick leave during the six months before the arthroplasty. The results of this study emphasize to use the CRTW model widely in collaboration between occupational health care providers and orthopedic clinics.

## Data Availability

The datasets generated and analyzed during the present study are not publicly available.
